# Autonomous Exploration of Unknown Indoor Environments for High-Quality Mapping Using Feature-Based RGB-D SLAM

**DOI:** 10.3390/s22145117

**Published:** 2022-07-07

**Authors:** Amr Eldemiry, Yajing Zou, Yaxin Li, Chih-Yung Wen, Wu Chen

**Affiliations:** 1 Department of Land Surveying and Geo-Informatics, The Hong Kong Polytechnic University, Hong Kong 999077, China; amr.eldemiry@connect.polyu.hk (A.E.); rick.zou@connect.polyu.hk (Y.Z.); yaxin-roan.li@polyu.edu.hk (Y.L.); 2 Shenzhen Research Institute, The Hong Kong Polytechnic University, Shenzhen 518057, China; 3 Department of Aeronautical and Aviation Engineering, The Hong Kong Polytechnic University, Hong Kong 999077, China; cywen@polyu.edu.hk

**Keywords:** autonomous exploration, mobile robots, 3D mapping quality, RGB-D SLAM, Voronoi planner

## Abstract

Simultaneous localization and mapping (SLAM) system-based indoor mapping using autonomous mobile robots in unknown environments is crucial for many applications, such as rescue scenarios, utility tunnel monitoring, and indoor 3D modeling. Researchers have proposed various strategies to obtain full coverage while minimizing exploration time; however, mapping quality factors have not been considered. In fact, mapping quality plays a pivotal role in 3D modeling, especially when using low-cost sensors in challenging indoor scenarios. This study proposes a novel exploration algorithm to simultaneously optimize exploration time and mapping quality using a low-cost RGB-D camera. Feature-based RGB-D SLAM is utilized due to its various advantages, such as low computational cost and dense real-time reconstruction ability. Subsequently, our novel exploration strategies consider the mapping quality factors of the RGB-D SLAM system. Exploration time optimization factors are also considered to set a new optimum goal. Furthermore, a Voronoi path planner is adopted for reliable, maximal obstacle clearance and fixed paths. According to the texture level, three exploration strategies are evaluated in three real-world environments. We achieve a significant enhancement in mapping quality and exploration time using our proposed exploration strategies compared to the baseline frontier-based exploration, particularly in a low-texture environment.

## 1. Introduction

The evolution of sensors and the robotics industry has attracted many researchers to focus on mobile robots. These robots can be mounted with onboard sensors to conduct indoor autonomous exploration missions. To name a few such missions, autonomous mobile robots can be exploited in rescue scenarios, utility tunnel monitoring, or indoor 3D mapping. Accordingly, all the required modules for autonomous exploration have been extensively studied. Such autonomous exploration modules in unknown environments using autonomous robots should model the scene from various connected locations to merge all local models into one consistent global map. Three main connected modules are needed to achieve autonomous mapping. First, a simultaneous localization and mapping (SLAM) system is required in GPS-denied environments, such as indoor environments. Several SLAM systems have been proposed, such as visual [1,2,3,4,5,6,7,8,9] and lidar SLAM systems [10,11,12,13,14]. Second, an exploration module that utilizes the obtained map and the localized robot is required to decide which location will be the next best goal, such as in the frontier-based exploration (FBE) [15] and next best view (NBV) approaches [16]. Finally, a path-planning module is needed to generate obstacle-free paths to reach the new goal, such as the A* path-planning algorithm first proposed in [17] or the rapidly exploring random trees (RRT) algorithm proposed in [18].

This study mainly focuses on exploration strategies and path planning to achieve high-quality mapping, especially in a low-texture environment. For the exploration module, the main question is, “Where is the best new goal?” This question should be answered according to the exploration’s main objectives: full coverage, the shortest exploration time, or the best 3D mapping quality. The “next goal” decision affects autonomous robots’ robustness to explore unknown environments safely and efficiently. However, several factors that challenge autonomous robots include the uncertainty in observations from the available sensors, imperfect control of robots, algorithm drawbacks, limited computational capability, limited payload, limited power consumption, and real-world complexity. Therefore, each module must be developed considering these factors, from the SLAM system to the exploration strategy and path planning. Furthermore, the integration of all modules should be considered to help them combine to produce a globally consistent high-quality map. For example, to achieve better mapping quality, the exploration strategy should consider the SLAM mapping quality requirements, such as the alignment accuracy and availability of loop closure, while selecting the next goal. In addition to the exploration strategy, the path-planning module is responsible for generating efficient, obstacle-free paths to reach the goal. However, to assist the SLAM system, these paths should have further characteristics such as smoothness and the same paths chosen between any two poses, back-and-forth. These characteristics help the loop closure detector find more accurate loops that enhance the SLAM drift, to generate a better, more consistent map.

Previous exploration strategies have not considered the mapping quality when using low-cost RGB-D sensors in a low-texture environment. To fill that gap, our study considered the mapping quality of such low-cost sensors in challenging scenarios (i.e., low-texture environments), seeking to improve their mapping quality. We developed a novel exploration system specially designed for feature-based RGB-D SLAM systems. The three main contributions of this study are summarized as follows:

1. A novel exploration strategy was developed using the number of features and their distribution uniformity score in 3D, thereby achieving better mapping quality using feature-based RGB-D SLAM.
2. A generalized Voronoi path planner was modified and implemented to keep the robot on a fixed road map of paths. Moreover, we ensured the same path was taken between any two positions (i.e., back-and-forth), to increase the probability of accurate loop closure detection. The robot also had the maximum clearance from any obstacle in the investigated, narrow real-world environments.
3. A comprehensive and intensive evaluation of our proposed autonomous exploration system was conducted in three real-world, complex, indoor scenarios, with its performance compared to the FBE approach.

This paper is organized as follows: Section 2 introduces the related works. The design and calculations for our proposed exploration system are explained in Section 3. Section 4 presents our experimental setup, results, and discussion. Finally, we draw conclusions in Section 5.

## 2. Related Works

The FBE approach [15] is the baseline exploration method as it is simple and has a low computational cost. The FBE is based on the concept of frontiers, i.e., the borders between free and unknown cells in a grid map. These frontiers are considered the gateways of map expansion. The robot can collect observations from its current location to build the starting map. This map size is limited according to the working range of the sensor used. Frontier detection can be applied to detect all frontiers beyond the threshold size. Subsequently, a new goal is chosen according to the nearest frontier, as proposed in [15]. This action, when repeated, should expand the overall map, and eventually come to reach all frontiers to cover the entire scene. However, the main disadvantage of this strategy is that it only considers the cost required to travel to the new goal selected, while ignoring mapping quality factors.

Alternatively, the NBV [16] is an exploration approach for selecting a new goal according to the maximum map expansion. Its new goal view should explore the largest possible space while also considering the travel cost. Goal candidates are generated and filtered according to their accessibility, the travel cost, and the expected area gain. This exploration approach may reduce the exploration time, but again, it does not consider the mapping quality of the implemented mapping system.

Most state-of-the-art approaches are based on FBE and NBV exploration methods but apply different optimization functions. Over the past few years, various exploration approaches [19,20,21,22] have been proposed to achieve different objectives. Cieslewski and Kaufmann [19] proposed a rapid exploration strategy based on FBE. The main objective of this rapid exploration approach is to explore at a non-zero velocity to avoid high power consumption by the unmanned aerial vehicle (UAV). This rapid exploration strategy adopts the FBE concept but only considers the frontiers in the current field of view. Thereafter, the frontier of the lowest cost according to the lowest velocity change is selected as the best new goal. In the case of no frontiers in the current field of view, the exploration approach switches to the classical frontier-based selection of the nearest frontier. This rapid exploration strategy enhances the UAV power consumption but still does not consider the mapping quality of low-cost sensors. Another FBE approach was proposed in [22], where the objective is to reduce the exploration time and path by applying an information gain function to choose the best frontier. However, this study once again ignored the mapping quality. Gomez and Hernandez [20] further proposed an FBE approach that applies semantic classification of the frontiers to reduce the exploration time and path, but as with the previous research, they did not consider the quality of the outputted 3D model. A histogram-based frontier exploration (HBFE) algorithm [23] was proposed to enhance the new goal selection by considering the distance and number of the frontier cells. This concept enabled the robot to select frontiers having a higher number of cells, assuming these frontiers have more extension mapping regions. The HBFE may decrease the exploration time, but the mapping quality is ignored. da Silva Lubanco and Pichler-Scheder [24] proposed an FBE algorithm to enhance the baseline FBE by applying a new utility function that considers motion cost, accessibility, and size for all frontiers to select the next new goal. These considered factors can enhance the exploration time of the baseline FBE while not considering the mapping quality factors. Beyond this, the efficient autonomous exploration approach proposed in [21] is designed for large-scale environments. The main concept is to combine FBE and NBV to avoid their drawbacks and take advantage of both methods. This exploration method works differently for local and global exploration missions. NBV is deployed as a local exploration approach, while FBE is utilized as a global exploration method. Elsewhere, Selin and Tiger [21] noted how NBV can be used to efficiently explore in small individual units but can easily get stuck in large environments without covering all regions. In contrast, FBE is better in large environments but suffers from unnecessarily moving back-and-forth between separate regions of the environment. The efficient autonomous exploration approach [21] the researchers applied reduced the exploration time in large-scale environments but did not consider the uncertainty around whether low-cost sensors can generate a consistent, high-quality map.

The feature-based RGB-D SLAM system is attractive due to its various advantages, such as a low computational cost, dense real-time reconstruction ability, and low-cost sensors. Consequently, this SLAM system has become popular for real-time indoor mapping and localization. On the other hand, high-end sensors and robots are utilized in extreme conditions, such as the exploration of the underground environments in the competitions funded by the Defense Advanced Research Projects Agency (DARPA), called the “DARPA Subterranean Challenge”. The last winning team [25] developed a team of robots to explore the three challenges of the competition: the “Tunnel Circuit”, the “Urban Circuit”, and the “Cave Circuit”, using their robot and exploration systems [26,27,28].

Contrary, the RGB-D sensor is cheap, has low power consumption, and can generate a dense map using its pixel-wise color and depth. Therefore, a feature-based RGB-D SLAM system was utilized for our exploration system. The mapping quality using feature-based RGB-D SLAM depends on two main factors. The first factor is the number of extractable features in the desired environment, and the second factor is the level of uniformity of these features. Therefore, the feature-based RGB-D SLAM system works well in texture-rich environments having evenly distributed features, while its performance is degraded in low-texture environments. Our proposed exploration approach accounts for these factors when selecting a new goal.

A path planner that maximizes the clearance between the robot and an obstacle should be considered for the following reasons: (1) to ensure obstacle avoidance in narrow and complex indoor environments, and (2) to guarantee the same path is taken back-and-forth between any two positions to increase the probability of detecting more accurate loop closures. Several path-planning algorithms have been proposed to generate efficient paths, such as search-based [17,29,30,31,32,33,34,35,36,37] and sampling-based planning methods [18,38,39,40,41,42,43,44]. Search-based algorithms such as the (A*) and A* variants [17,29,30,31,32,33,34,35,36,37] are designed to provide the optimal short path based on an evaluation function. This evaluation function provides the cost from the starting point to an adjacent node (n) and the cost from that node (n) to the goal point. In this way, the generated paths are not fixed, and the robot does not capture the same frame more than once. However, the generated paths increase the complexity of loop closure detection. Moreover, the clearance between the robot and obstacles is minimal, which lowers the safety in narrow environments. Further unresolved issues with this approach include sensor uncertainty and imperfect robot control. Sampling-based path-planning methods include rapidly exploring random trees (RRT) algorithms [18,38,39,40,41,42,43,44] that randomly expand trees to connect the starting point to the goal point. These trees are used to find the shortest path to the goal point, but consequently, the clearance between the robot and obstacles is minimal, which once again limits the safety in narrow environments.

Unlike the previous path-planning methods, the generalized Voronoi diagram (GVD) path planner [45,46,47] generates fixed paths for any fixed environment by detecting the paths with the maximum clearance from obstacles. As such, this path planner is the safer option for planning in narrow and complicated real-world environments compared to A* and RRT planners. For this reason, path fixation was of great importance for our exploration system to assist the SLAM with loop closure detection while mitigating the SLAM drift. Consequently, a GVD path planner [48] was modified and utilized in our proposed system. This modification (as mentioned in Section 3.3) was mainly proposed to force the robot to stay on the generated Voronoi paths rather than following a connection path to the Voronoi road map.

The above literature indicated that the mapping quality when using a low-cost sensor such as an RGB-D camera was not considered in most state-of-the-art exploration strategies. These strategies can be efficient in certain mapping conditions, such as in texture-rich environments, for perfect mapping, and to support localization systems. However, in real-world scenarios, such optimum conditions are not available; in that context, we propose exploration strategies to increase the mapping quality for challenging real-world scenarios. The proposed exploration strategies simultaneously achieve a high mapping quality and short exploration time in challenging conditions of real-world complexity, low texture, limited computational capability, SLAM drift, and uncertainty in the low-cost RGB-D observations.

## 3. Autonomous Exploration System

**Figure 1:**
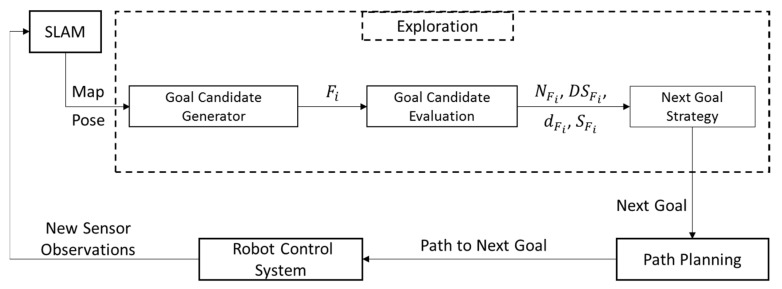
The architecture of the proposed exploration system, starting with SLAM to provide the occupancy map and the robot pose. The exploration module detects all frontiers ($F_{i}$) to be evaluated based on the number of features ($N_{F_{i}}$), distribution score ($DS_{F_{i}}$), distance to the robot ($d_{F_{i}}$), and size of the frontier ($S_{F_{i}}$). Accordingly, the next goal strategy selects the highest score candidate as the best new goal. The next goal is sent to the path planner to generate the path from the current robot position. Finally, the robot control system applies the received path to reach the next goal and gain new sensor observations. This exploration iteration is repeated until finishing all accessible frontiers.

This section presents our proposed approach to autonomous exploration—as shown in Figure 1—using a mobile robot mounted with an RGB-D sensor [49,50]. Our exploration method was developed to explore indoor environments, considering the parameters of mapping quality, complete coverage, and exploration time. FBE was adopted to generate the goal candidates [15,51]. A novel strategy was proposed that considers the mapping quality and exploration time to select the next best goal.

### 3.1. Generation of Goal Candidates

FBE [15] introduced the concept of frontiers, i.e., the borders between free and unknown cells in the grid map of an environment. According to our indoor mapping scenario, the robot can only detect its current location and the local map based on what it can observe from its current location. In our work, the first local map was based on the limitation of the utilized sensor’s working range and the robot motion type. Our system used TurtleBot2 [50], which can rotate in place to generate a starting circle map. This start map was used for the first iteration of frontier detection, to detect all frontiers beyond a threshold size. These frontiers were the possible targets considered as the next goal candidates for map expansion. Thereafter, these candidates were evaluated, as described in Section 3.2.

Frontier detection was implemented using a method proposed in [51] to detect the frontier cells. This greedy search method was applied to the available occupancy grid map built by the RGB-D SLAM system [9]. Each frontier cell was a known reachable free cell with an unknown cell adjacent to one of its edges. Equations (1) and (2) explain the frontier cell (F) detection mode. These equations are used to detect the frontier cell candidates, $C_{F}$, from all mapped cells, and the frontier cells (F) adjacent to unknown cells (U), respectively, as follows:

(1) $$C_{F}=\left\{C^{xy}|C^{xy}\in M_{free}\cap R\right\}$$

where $C_{F}$ represents the candidate frontier cells, $C^{xy}$ the 2D coordinates of all mapped cells, $M_{free}$ the free mapped cells, and R the reachable cells.

(2) $$F=\left\{C_{F}|\;C_{F}\exists \;U\right\}$$

where F represents the frontier cells, $C_{F}$ the frontier cell candidates, and U the unknown cells.

### 3.2. Evaluation of Goal Candidates

After every iteration of frontier detection, the influencing factors of mapping quality and exploration time were calculated for each detected frontier region. The area borders of every detected frontier were identified based on each frontier’s cell’s maximum and minimum 2D coordinates. According to these borders, all feature points extracted by SLAM were projected. Subsequently, the number of feature points ($N_{F_{i}}$) in every frontier area was calculated as follows:

(3) $$N_{F_{i}}=\left\{N\left(P\right)|\;P^{x}\in \left\{X_{min_{F_{i}}}\;,X_{max_{F_{i}}}\right\}\;⋀\;P^{y}\left\{Y_{min_{F_{i}}},Y_{max_{F_{i}}}\right\}\right\}$$

where $N\left(P\right)$ is the extracted feature number, $P^{x}\;\mathrm{and}\;P^{y}$ are the 2D coordinates of the current extracted feature points, and $X_{min_{F_{i}}}$, $X_{max_{F_{i}}}$, $Y_{min_{F_{i}}}$, and $Y_{max_{F_{i}}}$ are the minimum and maximum 2D coordinates of each frontier’s cells.

At this stage, each frontier was represented by the number of feature points ($N_{F}$) and their 3D coordinates. Further to this, a distribution uniformity score was calculated by adopting a chi-squared discrete uniform distribution test. The distribution score in the *x*-direction $DS^{x}$ is explained as follows:

(4) $$E_{i}=\frac{N_{F}}{n}$$

(5) $$DS^{x}=\overset{n}{\underset{i=1}{\sum }}\frac{\left(NP_{i}^{x}-E_{i}\right)}{E_{i}}$$

where $E_{i}$ is the expected number of feature points in each interval of the *x* direction for a discrete uniform distribution, $N_{F}$ is the total number of feature points projected in the frontier area, n is the total number of intervals according to the required interval size and the frontier’s *x* size, and $NP_{i}^{x}$ is the actual number of feature points in the interval *i*.

Using the same method, the distribution scores for the *y* and *z* directions ($DS_{F_{i}}^{y}\;\mathrm{and}\;DS_{F_{i}}^{z}$) were calculated to obtain the total distribution score of every frontier ($DS_{F_{i}}$) as follows:

(6) $$DS_{F_{i}}=-(DS_{F_{i}}^{x}+DS_{F_{i}}^{y}+DS_{F_{i}}^{z})$$

### 3.3. Next Goal Strategy

The baseline FBE [15] is a straightforward strategy that considers only the nearest accessible frontier to be the next goal. Therefore, the mapping quality is ignored in this strategy. On this basis, we proposed new score functions designed for feature-based RGB-D SLAM. The proposed strategies were based on the mapping quality factors besides the travel cost, to combine a high mapping quality with a short exploration time. The extracted feature points and the uniform distribution level are the main factors affecting the mapping quality when using feature-based RGB-D SLAM. Therefore, the proposed strategic concept was to explore the low-texture regions or unevenly distributed feature regions at the end of the exploration. This action increased the probability of extracting more features in these regions from more viewpoints and frames while exploring the better regions. In this way, the SLAM system can achieve a higher mapping quality in the same environment.

After filtering out frontiers of sizes less than the threshold size ($S_{min}$), the proposed cost function evaluated the frontiers with respect to the number of feature points and the distribution score of each frontier’s feature points. A minimum number of features ($N_{min}$) was used to postpone the frontiers of low feature points until the end of exploration. Equations (7)–(9) describe three exploration strategies. The first strategy described by Equation (7) is the baseline strategy (D strategy). The second strategy described by Equation (8) is the proposed mapping quality strategy (M strategy). Finally, Equation (9) shows the combined strategy (M+D strategy), which combines the mapping quality and travel cost factors.

D strategy:

(7) $$GS_{F_{i}}=-d_{F_{i}}$$

M strategy:

(8) $$\left\{\begin{array}{c} \mathrm{for}\;N_{F_{i}}<N_{min},\;GS_{F_{i}}=-\infty \\ \mathrm{for}\;N_{F_{i}}\;\geq \;N_{min},\;GS_{F_{i}}=N_{F_{i}}+DS_{F_{i}} \end{array}\right.$$

M+D strategy:

(9) $$\left\{\begin{array}{c} \mathrm{for}\;N_{F_{i}}<N_{min},GS_{F_{i}}=-\infty \\ \mathrm{for}\;N_{F_{i}}\;\geq \;N_{min},GS_{F_{i}}=N_{F_{i}}+DS_{F_{i}}-d_{F_{i}} \end{array}\right.$$

where $GS_{F_{i}}$ is the goal score of the frontier, $d_{F_{i}}$ is the distance between the robot and the frontier, $N_{F_{i}}$ is the number of features in the frontier’s region, $N_{min}$ is the minimum accepted number of features in a frontier’s region, and $DS_{F_{i}}$ is the distribution score of features in the frontier’s region.

### 3.4. Path Planning

A generalized Voronoi diagram path planner [47] generates a fixed road map for any fixed environment by detecting all Voronoi edges ($E_{ij}$) between any two obstacles, i and j, as follows:

(10) $$E_{ij}=\left\{x\in {\mathbb{R}}^{2}|d\left(x,f_{i}\right)=d\left(x,f_{j}\;\right),\;\leq d\left(x,f_{k}\right),\;for\;all\;k\neq i,\;j\;\right\}$$

where x is the point of the Voronoi edge ($E_{ij}$) detected in the center between any two obstacles.

The generated paths maximized the clearance between the robot and obstacles and included fixed paths for the same fixed environment. This path fixation ensured the same frames were observed between the same two positions. Furthermore, we modified the GVD planner [47]. The modification was made to neglect a segment outside the generated Voronoi road map. The GVD planner generates three segments for every path. The first segment is a path between the robot’s position and the nearest point on the Voronoi edges. The second segment is a path between the target point and the nearest point on the Voronoi edges. Finally, the GVD planner detects the shortest path within the Voronoi road map connecting the last two segments. In our work, we modified the second segment to only detect the nearest point in the Voronoi road map to the target point and not generate the second segment’s usual path to the target point. Consequently, the robot only traveled to the fixed Voronoi edges to observe the same frames, meaning the SLAM loop closing module could detect more accurate loops to mitigate SLAM drift.

## 4. Experiments and Discussion

This section introduces the experimental setup and results, including the hardware and software we utilized and the experimental scenarios. Moreover, the discussion around each experiment is introduced.

### 4.1. Hardware

**Figure 2:**
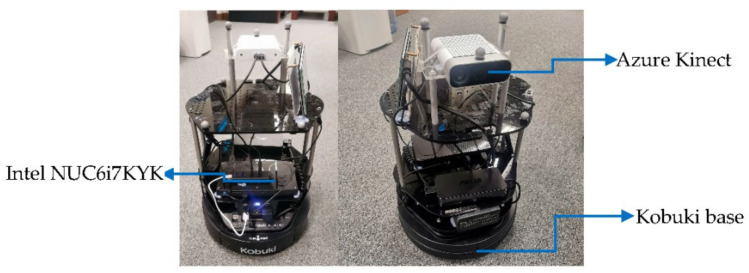
Front and back views of the TurtleBot2 with Kobuki base, Azure Kinect RGB-D sensor mounted on the top, and Intel NUC6i7KYK Mini PC.

For all experiments in real-world environments, we used TurtleBot2 with the Kobuki base [50] as a mobile robot mounted with an RGB-D sensor (Azure Kinect) [49], and we added a mini-PC (Intel NUC6i7KYK) [52], as shown in Figure 2.

### 4.2. Software

Our feature-based RGB-D SLAM module [9] was utilized for mapping and localization. The proposed exploration system was implemented based on explore_lite [53], an open-source frontier-based ROS package. The new proposed mapping factors and strategies were added to this ROS package. The modified Voronoi path planner was implemented based on the open-source voronoi_planner package [48], a global planner in the move_base navigation ROS package [54].

The mapping quality was evaluated according to the point-to-point distances (PTPDs) between the points of the ground truth 3D models and the points of the output model of each experiment after point cloud registration [55,56,57,58] for the two models. The loop closing method was according to our feature-based RGB-D SLAM system (in [9], Section 5.2).

### 4.3. Low-Texture Experiment

Three exploration strategies were investigated, as mentioned in Section 3.3. The “D strategy” was the classical FBE that considered only the distance between the robot and the next goal candidates to decide the next goal. The second proposed “M strategy” considered feature point numbers and distribution scores. The last proposed “M+D strategy” involved combining the classical nearest goal strategy with the mapping strategy. Three trials of every exploration strategy were implemented to investigate the enhancement rates for each of the two proposed strategies compared to the baseline D strategy.

**Figure 3:**
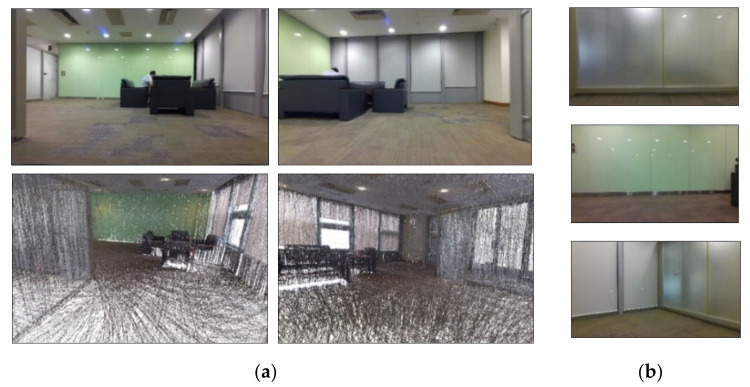
Low-texture setup. (**a**) The upper two images show the surveying room, and the lower two show the ground truth model using a Leica BLK360 imaging laser scanner. (**b**) Samples of the observed low-texture frames.

The low-texture surveying scene is shown in Figure 3. Its dimensions are 7.5 m-long and 6.5 m-wide. As shown in Figure 3, this scene has multiple clear walls with few features and a sofa in only one corner of the room. The ground truth was collected using a Leica BLK360 [59], a high-accuracy imaging laser scanner.

We calculated the PTPD between each experimental trial’s aligned output point cloud and the ground truth point cloud, to evaluate the mapping quality.

Table 1 shows that our proposed exploration strategies M and M+D clearly achieved a better output for the 3D model. The PTPD RMSE and STD were enhanced by more than 40% compared to the baseline exploration D strategy output. Furthermore, the total path length and total exploration time were less than those of the D strategy by nearly 30% and 23% for both the M strategy and the M+D strategy, respectively. Our proposed exploration strategies also increased the number of detected loop closures from zero in two trials of the D strategy to always having loop closures, reaching 18 detected loops, in the M and M+D strategies.

**Figure 4:**
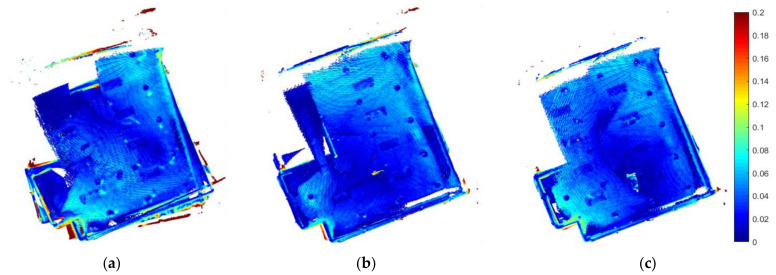
Low-texture PTPD (m) between exploration models and the ground truth model. (**a**) D strategy, (**b**) M strategy, and (**c**) M+D strategy.

**Figure 5:**
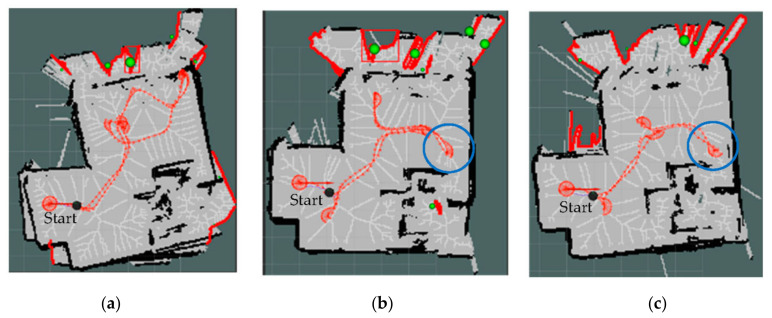
Exploration paths of the low-texture environment, the bold red clusters are the ignored inaccessible frontiers, and the green balls represent the centroids of the frontiers. (**a**) D strategy, (**b**) M strategy, and (**c**) M+D strategy.

Figure 4 shows the PTPD enhancement levels in our proposed exploration strategies M and M+D. It is obvious that the baseline strategy had more PTPD greater than 18 cm and misalignments in its mapping. Further to this, Figure 5 introduces the paths of the three exploration strategies. As can be seen from the blue circles in Figure 5b,c, the paths of the proposed strategies (M and M+D) tended to first explore the texture-rich area in the room, while the baseline strategy ignored the texture-rich region. Therefore, the D strategy observed more low-texture frames at the start of the exploration, which degraded the mapping quality. The bold red lines in Figure 5 are inaccessible frontiers outside the room that could be observed as the walls were made of glass.

### 4.4. Moderate-Texture Experiment

**Figure 6:**
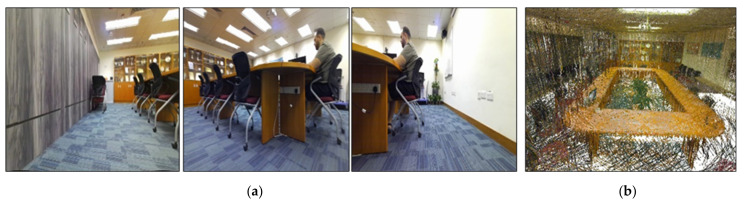
Moderate-texture setup. (**a**) The three left frames show the surveying environment containing some low-texture walls. (**b**) Ground truth model using the Leica BLK360 imaging laser scanner.

In the moderate-texture environment, there were low-texture walls, as can be seen in Figure 6, and a large table in the center of the room provided more features to observe in the frames. In the case of the robot facing the white walls, the observed frames suffered from the lack of features. The room dimensions were 8.5 m-long and 7.5 m-wide. The 3D point cloud ground truth was collected using the same high-accuracy imaging laser scanner (Leica BLK360) [59].

Table 2 shows that our proposed exploration strategies enhanced the PTPD RMSE by 15.6% for the M strategy and 13.5% for the M+D strategy compared to the D strategy. Furthermore, there was a near 10% enhancement in PTPD STD for both proposed strategies. However, the total exploration path length was increased from just under 35 m in the D strategy to 45 m in the M strategy, while in the M+D strategy, the total exploration path length was in the same range as the D strategy. The robot increased the back-and-forth travel in the M strategy as the SLAM system updated the extracted features while reaching the current goal. Therefore, the total exploration time increased from 212 s in the D strategy to 260 s in the M strategy. The number of detected loop closures increased dramatically from nearly zero in the D strategy to 36 in the M strategy and 20 in the M+D strategy.

**Figure 7:**
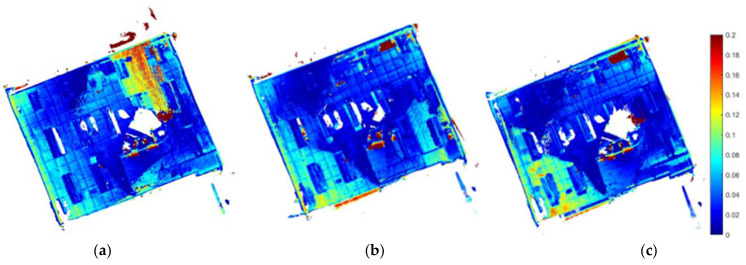
Moderate-texture PTPD (m) between exploration models and the ground truth model. (**a**) D strategy, (**b**) M strategy, and (**c**) M+D strategy.

**Figure 8:**
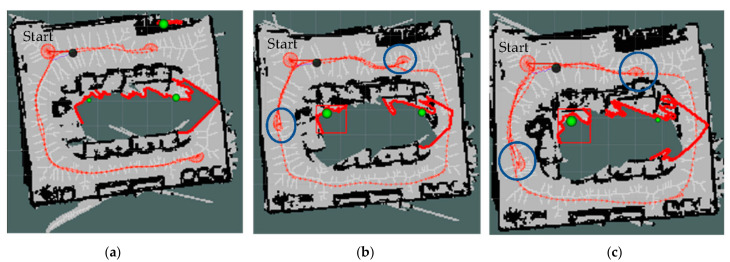
Exploration paths of the moderate-texture environment, the bold red clusters are the ignored inaccessible frontiers, and the green balls represent the centroids of the frontiers. (**a**) D strategy, (**b**) M strategy, and (**c**) M+D strategy.

Figure 7 shows the PTPD for the moderate-texture environment, with lower PTPD in both the M and M+D strategies compared to the D strategy. Figure 8 shows the exploration paths of all strategies, with blue circles in Figure 8b,c. These circles show the turns that increased the probability of detecting more loop closures in our proposed M and M+D strategies.

### 4.5. Texture-Rich Experiment

**Figure 9:**
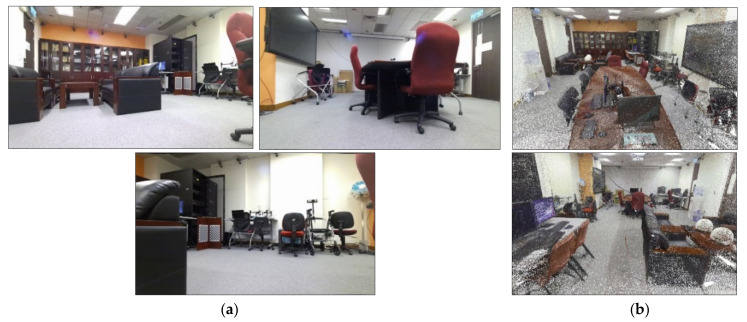
Texture-rich setup. (**a**) The left three frames show the surveying environment containing some texture-rich regions. (**b**) Ground truth model using the Leica RTC360 3D laser scanner.

The last environment had rich feature points in most observed frames, as shown in Figure 9a. The dimensions of this room were 10 m-long and 6 m-wide. The 3D point cloud ground truth (as shown in Figure 9b) was collected using a high-accuracy 3D laser scanner (Leica RTC360) [60].

Table 3 shows that our proposed exploration strategies did not significantly enhance the mapping of the texture-rich environment, which is reasonable. Almost all RMSEs of the PTPD were within the range of 5.5–6 cm, but the total path length and the total exploration time were enhanced by nearly 26% and 34% for the M and M+D strategies, respectively. In a texture-rich environment, the mapping quality using feature-based SLAM should always be high, regardless of the exploration strategy. Subsequently, the number of detected loop closures was enough for all strategies, thus enabling SLAM to efficiently mitigate the drift each time.

**Figure 10:**
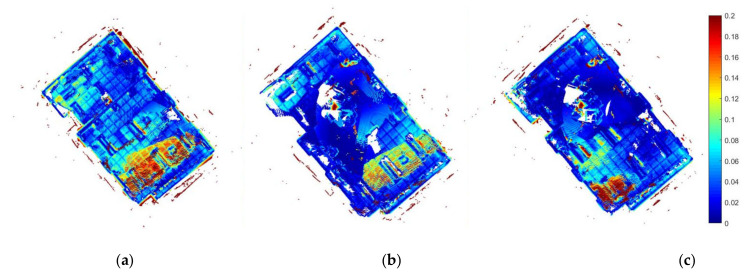
Texture-rich PTPD (m) between exploration models and the ground truth model. (**a**) D strategy, (**b**) M strategy, and (**c**) M+D strategy.

**Figure 11:**
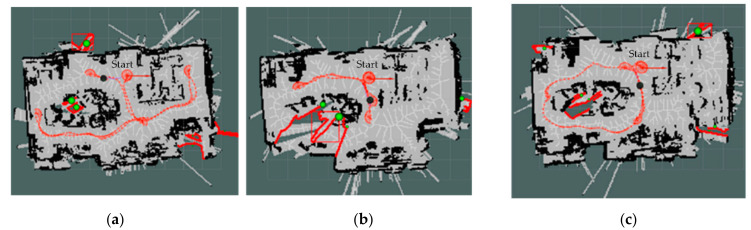
Exploration paths of the texture-rich environment, the bold red clusters are the ignored inaccessible frontiers, and the green balls represent the centroids of the frontiers. (**a**) D strategy, (**b**) M strategy, and (**c**) M+D strategy.

As a result of all regions in the texture-rich environment having sufficient features, the mapping quality using feature-based SLAM was high for all exploration strategies. PTPDs are shown in Figure 10, which indicates that all models have nearly the same accuracy, despite taking different exploration paths, as shown in Figure 11.

## 5. Conclusions

To obtain a better mapping quality using a feature-based RGB-D SLAM system for autonomous exploration in indoor real-world environments, we proposed a novel autonomous exploration system to assist SLAM to build better-quality maps. Our concluding remarks are as follows:

1. A GVD path planner was modified to keep the robot on a fixed road map to increase the probability of accurate loop closure detection.
2. A novel autonomous exploration strategy was proposed specifically for feature-based RGB-D SLAM. The number of features and their distribution were considered to obtain better mapping quality.
3. The proposed autonomous exploration system was evaluated in three real-world indoor environments (chosen according to the availability of features). Our evaluation concerned how the mapping quality was enhanced compared to the baseline FBE strategy. To that end, we tested the baseline classical frontier-based strategy (D strategy), our proposed mapping quality strategy (M strategy), and a combination of the two (M+D strategy). The results may be summarized as follows:
  - In the low-texture environment, we achieved a high mapping quality, which indicates that when using feature-based SLAM, it is valuable to consider the number of features and their distribution to decide the next goal. The enhancement in mapping quality was significant, i.e., more than 40% in PTPD RMSE for both the M and M+D strategies compared to the D strategy. Moreover, the total path length and exploration time were reduced by nearly 30% and 23% for the M and M+D strategies, respectively, compared to the D strategy.
  - In the moderate-texture environment, the enhancement in mapping quality was 15.6% in PTPD RMSE for the M strategy and 13.5% for the M+D strategy. The total exploration path length increased from under 35 to 45 m for the M strategy, but it remained in the same range for the M+D strategy.
  - In the texture-rich environment, our strategy only slightly enhanced the mapping quality, i.e., by less than 3% in PTPD RMSE for the M strategy and 10% for the M+D strategy. However, the total path length and exploration time were enhanced by 26% and 34% for the M and M+D strategies, respectively.

The results indicate that our proposed exploration strategy dramatically enhanced the mapping quality and reduced the total exploration time and total path length in the challenging low-texture environment. At the other end of the spectrum, the mapping was high-quality in the texture-rich environment for all strategies, as in such texture-rich environments, feature-based SLAM systems can always detect many features that increase the alignment accuracy and the mapping quality, regardless of the exploration strategy.

Future work should consider other SLAM systems and design specific exploration strategies for these. Moreover, an exploration strategy should be proposed for online mapping quality estimation, to ensure the required mapping accuracy has been achieved before the exploration ceases.

## Figures and Tables

**Table 1:** Low-texture results. PTPD RMSE (cm), PTPD STD (cm), number of loop closures (LC), exploration path length (L) (m), and exploration time (T) (s).

Strategy_trial	RMSE (cm)	STD (cm)	#LC	L (m)	T (s)
D_1	8.94	6.80	0	35.08	191.3
D_2	10.36	7.70	0	31.14	179.1
D_3	7.95	5.91	11	69.88	436.1
Average	9.08	6.80	3.7	45.37	268.8
M_1	6.02	4.74	2	23.74	140.6
M_2	4.96	3.80	7	37.57	206.2
M_3	4.83	3.61	4	36.44	186.4
Average	5.27	4.05	4.3	32.59	177.7
Enhancement	41.9%	40.5%	+0.6 loops	28.2%	33.9%
M+D_1	5.06	4.03	18	43.07	258.9
M+D_2	4.90	3.66	3	27.25	154.9
M+D_3	5.12	4.21	11	36.63	200.8
Average	5.03	3.97	10.7	35.65	204.9
Enhancement	44.6%	41.7%	+7 loops	21.4%	23.8%

**Table 2:** Moderate-texture results. PTPD RMSE (cm), PTPD STD (cm), number of loop closures (LCs), exploration path length (L) (m), and exploration time (T) (s).

Strategy_trial	RMSE (cm)	STD (cm)	#LC	L (m)	T (s)
D_1	7.58	5.82	0	33.89	204.0
D_2	8.92	6.58	0	34.29	206.6
D_3	6.76	5.31	2	34.92	225.3
Average	7.75	5.90	0.7	34.37	212.0
M_1	6.88	5.54	50	51.98	297.2
M_2	6.67	5.24	36	38.98	238.1
M_3	6.09	4.82	22	42.13	244.7
Average	6.55	5.20	36	44.36	260.0
Enhancement	15.6%	12%	+35 loops	−29%	−22%
M+D_1	6.96	5.60	27	37.33	220.3
M+D_2	6.30	4.98	2	34.85	214.3
M+D_3	6.86	5.36	31	42.90	243.2
Average	6.71	5.31	20	38.36	225.9
Enhancement	13.5%	10.0%	+19 loops	−11%	−6%

**Table 3:** Texture-rich results. PTPD RMSE (cm), PTPD STD (cm), number of loop closures (LCs), exploration path length (L) (m), and exploration time (T) (s).

Strategy_trial	RMSE (cm)	STD (cm)	#LC	L (m)	T (s)
D_1	5.74	4.25	12	30.46	191.0
D_2	6.37	4.51	8	40.18	266.7
D_3	5.79	4.49	4	36.70	240.0
Average	5.97	4.42	8	35.78	232.6
M_1	6.04	4.65	17	37.88	238.6
M_2	4.16	3.22	13	19.21	117.7
M_3	7.33	5.21	10	21.54	160.3
Average	5.84	4.36	13.3	26.21	172.2
Enhancement	2.1%	1.3%	+5 loops	26.7%	26.0%
M+D_1	5.44	4.10	4	13.84	90.8
M+D_2	5.30	4.02	15	29.69	192.5
M+D_3	5.42	4.15	11	27.33	175.3
Average	5.39	4.09	10	23.62	152.9
Enhancement	9.7%	7.4%	+2 loops	34.0%	34.3%

## Data Availability

Not applicable.
